# Uremic Retention Solute Indoxyl Sulfate Level Is Associated with Prolonged QTc Interval in Early CKD Patients

**DOI:** 10.1371/journal.pone.0119545

**Published:** 2015-04-20

**Authors:** Wei-Hua Tang, Chao-Ping Wang, Fu-Mei Chung, Lynn L. H. Huang, Teng-Hung Yu, Wei-Chin Hung, Li-Fen Lu, Po-Yuan Chen, Ching-Hsing Luo, Kun-Tai Lee, Yau-Jiunn Lee, Wen-Ter Lai

**Affiliations:** 1 Graduate Institute of Medicine, Collage of Medicine, Kaohsiung Medical University, Kaohsiung, Taiwan; 2 Division of Cardiology, Department of Internal Medicine, Kaohsiung Medical University Hospital, Kaohsiung Medical University, Kaohsiung, Taiwan; 3 Division of Cardiology, Department of Internal Medicine E-Da Hospital, I-Shou University, Kaohsiung, Taiwan; 4 Institute of Biotechnology, National Cheng Kung University, Tainan, Taiwan; 5 Division of Cardiac Surgery, Department of Surgery, E-Da Hospital, I-Shou University, Kaohsiung, Taiwan; 6 Institute of Electric Engineering, National Cheng-Kung University, Tainan, Taiwan; 7 Lee’s Endocrinologic Clinic, Pingtung, Taiwan; University of Florida, UNITED STATES

## Abstract

Total mortality and sudden cardiac death is highly prevalent in patients with chronic kidney disease (CKD). In CKD patients, the protein-bound uremic retention solute indoxyl sulfate (IS) is independently associated with cardiovascular disease. However, the underlying mechanisms of this association have yet to be elucidated. The relationship between IS and cardiac electrocardiographic parameters was investigated in a prospective observational study among early CKD patients. IS arrhythmogenic effect was evaluated by in vitro cardiomyocyte electrophysiological study and mathematical computer simulation. In a cohort of 100 early CKD patients, patients with corrected QT (QTc) prolongation had higher IS levels. Furthermore, serum IS level was independently associated with prolonged QTc interval. In vitro, the delay rectifier potassium current (IK) was found to be significantly decreased after the treatment of IS in a dose-dependent manner. The modulation of IS to the IK was through the regulation of the major potassium ion channel protein Kv 2.1 phosphorylation. In a computer simulation, the decrease of IK by IS could prolong the action potential duration (APD) and induce early afterdepolarization, which is known to be a trigger mechanism of lethal ventricular arrhythmias. In conclusion, serum IS level is independently associated with the prolonged QTc interval in early CKD patients. IS down-regulated *I_K_* channel protein phosphorylation and the *I_K_* current activity that in turn increased the cardiomyocyte APD and QTc interval in vitro and in the computer ORd model. These findings suggest that IS may play a role in the development of arrhythmogenesis in CKD patients.

## Introduction

The importance of cardiovascular diseases (CVD) and cardiac arrhythmias in chronic kidney disease (CKD) is currently well established. Abnormal corrected QT (QTc) prolongation on the electrocardiogram, which is an independent risk factor for sudden cardiac death (SCD), is frequently found in patients with CKD and end-stage renal disease (ESRD) [1,2]. However, traditional cardiovascular risk factors are insufficient to explain and accurately predict QTc prolongation and SCD in this population. As the renal function deteriorates, levels of uremic retention toxins and proinflammatory cytokines increase [3]. Various inflammatory mediators, such as C-reactive protein (CRP), IL-6 and platelet-activating factor, have been associated with arrhythmias through the modulation of ion channel function [4,5]. In CKD patients, the protein bound uremic retention solutes, such as Indoxyl sulfate (IS), is associated with the elevation of inflammatory mediators [6,7], elevation of serum IL-6 [8], anti-oxidant modulation [6,7] and monocyte-mediated inflammation [9]. Our previous studies have also demonstrated that increased serum IS may be a possible risk factor in the pathogenesis of coronary atherosclerosis in CKD patients [10,11]. However, the association between IS levels and the cardiac arrhythmias risk indicator remains was still unknown. In the present study, we thus examined the relationship of serum IS levels with electrocardiographic parameters in a cohort of early CKD patients.

The delayed rectifier potassium current (*I*_*K*_) plays an important role in the repolarization of cardiac action potentials and is one of the major currents that determines the action potentials duration and QT interval [12,13]. The potassium channel trafficking and regulation are involved in certain inherited or acquired cardiac channelopathies, such as long QT syndrome and heart failure [14]. For these reasons, the IS effect on cardiomyocyte electrophysiology was explored in vitro and in a mathematical computer simulation model in the present report.

## Materials and Methods

### Participants

The study investigated 100 consecutive CKD patients who underwent cardiac multi-slice CT or coronary angiography for exclusion of CAD due to typical and atypical chest pain, with intermediate pretest indications for CAD, from June 2006 to June 2008 at the E-Da Hospital. Before the examination, all of the each patients received a detailed interview covering their medical and personal histories. Patients with previous diagnoses of cerebrovascular diseases, heart failure, cardiomyopathy, coronary heart diseases, congenital heart disease, cardiac arrhythmias or taking medication that had a QT prolongation effect [15] were excluded from this study. Type 2 diabetes (T2DM) was defined as a past or current diagnosis of T2DM and/or the need for medical therapy. Hypertension was defined as a systolic blood pressure (SBP) ≥ 140 mmHg, a diastolic blood pressure (DBP) ≥ 90 mmHg, or if the patient was under antihypertensive treatment. Dyslipidemia was defined according to the criteria of Adult Treatment Panel III, or if the patient was under lipid lowering treatment. Former and current smokers were analyzed as one group, and compared with those who had never smoked.

Estimated glomerular filtration rate (eGFR) had been calculated and followed up according to the extended Modification of Diet in Renal Disease Study formula 3–6 months before the study [16]. Written informed consent was obtained before the enrollment. This study was approved by the Human Research Ethics Committee of I-Shou University E-Da Hospital.

### Laboratory measurement

Peripheral blood samples were taken after fasting for 8 hours overnight and before the examination. Complete blood counts and serum creatinine, sodium, potassium, calcium, uric acid, albumin, glucose and lipid profiles (included plasma triglycerides, total cholesterol, LDL-C, HDL-C) were determined as our previous reports [17,18].

To determine the blood total IS, A UPLC assay was used as our previously reported [10,17,18]. In brief, the blood for total IS determination was drawn, centrifuged, and stored at-80°C for subsequent assay immediately after blood sampling. The serum samples were deproteinized by the addition of 3 parts methanol to 1 part serum for determination of IS. A UPLC assay, using detection at the 280 nm of the PDA detector, was performed at room temperature on an ACQULITY UPLC BEH phenyl column of 2.1 × 100 mm. Quantitative results were obtained and calculated as concentrations (μmol/L). The sensitivity of this assay was 1.061 μmol/L for IS.

### ECG, QT and QTc interval measurement

Twelve-lead ECGs were collected at the baseline examination by standardized protocol. Standard interval (heart rate, PR, QRS, QT intervals), amplitudes (R, S, and T waves and J and ST segment) were analyzed by standard protocol as described elsewhere [19]. The QT and QTc interval were calculated by the post-processing of ECG signals using the superimposed median beat method and Bazett's formula (QTc = QT/ √RR). QRS interval with a bundle branch block pattern, duration longer than 120 ms, extremely rapid (> 150 beats per minute) or extremely slow heart rate (< 40 beats per minute) ECG were excluded from this study [20,21]. The definition of prolonged QTc interval in our study was according to the 2009 AHA/ACCF/HRS recommendations. The adjusted QT of 460 ms or longer in women and 450 ms or longer in men was considered a prolonged QT interval [22].

### H9c2 cell culture and IS treatment

Embryonic rat heart-derived cardiac H9c2 cells (BCRC 60096, Bioresource Collection and Research Center, Taiwan) were cultured in DMEM supplemented with 10% fetal bovine serum under an atmosphere of 95% air 5% CO2 at 37°C. Experiments were carried out using mononucleated myoblasts culture for 2–5 days [23]. All culture media were controlled to pH 7.4 before use. The cells were incubated 48 hours with IS (purchased from Sigma) at a concentration of 0.1μM, 1μM and 300μM, as previously described [24], before the experiment. In addition, we did not investigate the effect of albumin and potassium in the present study for the following reasons. Meijers et al. revealed that the presence of albumin, even in abnormal high concentration, still did not significantly affect the protein-bound uremic toxin biological effect [25]. Further, in this study because *p*-cresyl sulfate was synthesized as a potassium salt, cells exposed to culture medium supplemented with equimolar concentrations of potassium chloride were used as controls. We found that the addition of 1.0 mmol/L of potassium chloride did not alter the number of EMPs compared with growth medium alone [25].

### Western blot analysis

The protein level of Kv2.1 and phosphorylated Kv2.1 in H9c2 cells was analyzed by Western blot [26,27]. In brief, after the total protein content was extracted, separated and transferred to Immobilon PVDF membranes (Millipore Corp., California, USA), rabbit polyclonal antibodies for Kv 2.1 (Millipore Corp., California, USA) or rabbit polyclonal antibodies for phosphorylated-Ser805 Kv2.1 (Sigma, USA) were added and incubated at room temperature. A secondary antibody (anti-rabbit, Millipore Corp., California, USA) conjugated with horseradish peroxidase was added, and the antigen-antibody complexes were detected by enhanced chemiluminescence (Millipore Corp., California, USA). Densitometric analysis was conducted using LabWorks 4.5 Image Acquisition and Analysis software (Ultra-Violet Products Ltd., UK).

### Patch-clamp cell electrophysiological studies

The whole-cell potassium outward currents were recorded using an Axopatch 700A amplifier (Axon Instruments, Union City, CA, USA). The details of the methods has been described in previous reports [23,28,29]. Briefly, H9c2 cells were placed in a recording dish and perfused with a bath solution and the cells were voltage clamped. Step-pulse protocols and data acquisition were performed using pCLAMP software (Axon Instruments). Membrane capacitance was calculated from the peak amplitudes and time constant decay of capacity transients elicited by 10 mV, hyperpolarizing voltage pulses from holding potential of -50 mV. All electrical recordings were performed at room temperature.

### Mathematical computer model for cardiomyocyte action potential and pseudo-ECG

The latest mathematical model of the O'Hara-Rudy dynamic human ventricular model (ORd model) was used in our experiment [30]. Cardiomyocyte action potential was mathematically constructed to include ionic currents, ionic pumps, ionic exchangers, and intracellular ionic regulation processes of Na^+^, K^+^ and Ca^2+^.

A Markov model for *I*_*K*_ is derived from previously published K channel models [31]. The current traces figures and data of *I*_*K*_ in H9c2 cells with and without IS treatment was digitized and formulated into a new Markov model computer equation and inserted into the ORd model to evaluate the IS effect on human cardiomyocyte action potential.

A pseudo-ECG was constructed and simulated by the transmural wedge model [32]. The numerical method of forward Euler with the Rush and Larsen method was used to compute pseudo-ECG with an integration time-step size (0.005ms) [33].

### Statistical analysis

Data normality was analyzed using the Kolmogorov-Smirnov test. Continuous, normally distributed variables are presented as mean ± SD, and non-normally distributed variables as median (interquartile range). Statistical differences in variables were compared using the Wilcoxon rank-sum test. Categorical variables were recorded as frequencies and/or percentages, and inter-group comparisons were analyzed with the Fisher’s exact test. The general linear modeling function analysis was used to control for potential confounders other than age and sex. Simple linear regression analysis was used to examine the association and independence between serum IS and the values of other parameters. Using multiple logistic regression, variables were assessed for independent associations with the prolonged QTc interval. Multivariate adjusted ORs are presented with 95% confidence interval (CI). All the tests were two-tailed, and a p value of < 0.05 was considered statistically significant. All of the statistical analyses were performed using SAS statistical software, version 8.2 (SAS Institute Inc., Cary, NC, USA).

## Results

### Clinical characteristics

The general demographics of the participants are summarized in Table 1. All the participants were with stage 2 or 3 CKD. The average eGFR was 63.0 ± 14.6 ml/min/1.73m^2^ (Table 1). All the patients in our studies were found with normal coronary angiogram over the cardiac multi-slice CT or coronary angiography. Fifty-six percent of our patients were male. The mean age of our patients is 61 ± 10 years and the average body mass index of our patients was 25.2 ± 3.6 kg/m^2^. Sixty-five percent of the patients had hypertension, 22% had diabetes, and 53% were diagnosed with hyperlipidemia, and 32% admitted to the use of tobacco.

**Table 1:** Patient demographics of 100 chronic kidney disease patients.

Age (years)	61 (54–69)
Men/Women (n, %)	56/44 (56/44)
Hypertension (% yes/no)	65/35
Diabetes mellitus (% yes/no)	22/78
Hyperlipidemia (% yes/no)	53/47
Current smoking (% yes/no)	32/68
Body mass index (kg/m^2^)	25.2 ± 3.6
Systolic blood pressure (mmHg)	129 ± 19
Diastolic blood pressure (mmHg)	75 ± 11
Fasting sugar (mg/dl)	111.2 ± 37.3
Total cholesterol (mg/dl)	181.0 ± 41.6
Triglyceride (mg/dl)	154.7 (77.5–173.5)
HDL-cholesterol (mg/dl)	44.8 ± 12.9
LDL-cholesterol (mg/dl)	106.1 ± 37.2
Hematocrit (%)	40.3 ± 4.6
Creatinine (mg/dl)	1.4 ± 0.9
GFR-MDRDGFR-E (ml/min/1.73m^2^)	63.0 ± 14.6
Albumin (mg/dl)	4.1 ± 0.3
Indoxyl sulfate (μmol/L)	6.1 (0.9–6.1)
Hs-CRP (mg/L)	4.8 (0.8–4.0)
Electrocardiographic parameters	
Rate (bpm)	73.0 ± 16.3
PR interval (ms)	161.5 ± 24.2
QRS duration (ms)	94.0 ± 17.3
QT interval (ms)	401.8 ± 44.6
QTc interval (ms)	436.7 ± 40.4

Values expressed as number (percent), mean ±SD, or median (25th to 75th percentile), as appropriate. Bpm: beats per minute, HDL: high-density lipoprotein, LDL: low-density lipoprotein, Hs-CRP: high-sensitivity C-reactive protein.

The median serum IS level was 6.1 μmol/L and the median of high sensitive CRP was 4.8 mg/L. The average heart rate was 73.0 ± 16.3 bpm, PR interval 161.5 ± 24.2ms, QRS duration 94.0 ± 17.3ms, QT interval 401.8 ± 44.6ms and QTc interval 436.7 ± 40.4ms.

### Association between serum IS and patients’ clinical characteristics

A univariate analysis was performed to test the association between the clinical and biochemical variables with log-transformed serum IS levels (Table 2). Serum IS was found positively associated with the age, creatinine, QRS duration, QT and QTc interval. In contrast, serum IS concentration was inversely associated to hematocrit, albumin, and eGFR.

**Table 2:** Clinical and biochemical variables associated in univariate analysis with log indoxyl sulfate.

Variable	Unit	β Coefficient (confidence interval)	p value
Age	year	0.329 (0.006 to 0.023)	0.001
Sex	Men *v* women	0.115 (-0.076 to 0.286)	0.253
Hypertension	Yes (65) *v* no (35)	0.057 (-0.135 to 0.243)	0.573
Diabetes mellitus	Yes (22) *v* no (78)	0.182 (-0.017 to 0.412)	0.070
Hyperlipidemia	Yes (53) *v* no (47)	0.017 (-0.166 to 0.196)	0.868
Current smoking	Yes (32) *v* no (67)	0.059 (-0.136 to 0.249)	0.561
Body mass index	kg/m^2^	-0.124 (-0.041 to 0.010)	0.218
Systolic blood pressure	mmHg	0.048 (-0.004 to 0.006)	0.633
Diastolic blood pressure	mmHg	-0.171 (-0.016 to 0.001)	0.088
Fasting sugar	mg/dl	0.031 (-0.002 to 0.003)	0.770
Total cholesterol	mg/dl	-0.160 (-0.004 to 0.000)	0.116
Triglyceride	mg/dl	0.037 (-0.269 to 0.391)	0.714
HDL-cholesterol	mg/dl	-0.070 (-0.009 to 0.005)	0.500
LDL-cholesterol	mg/dl	-0.201 (-0.005 to 0.000)	0.051
Hematocrit	%	-0.237 (-0.045 to -0.003)	0.025
Creatinine	mg/dl	0.524 (0.954 to 1.878)	<0.001
Estimated GFR	ml/min/1.73m^2^	-0.521 (-0.021 to -0.009)	<0.001
Albumin	mg/dl	-0.273 (-0.629 to -0.050)	0.022
Hs-CRP	mg/L	-0.067 (-0.018 to 0.010)	0.580
ECG rate	bpm	0.102 (-0.003 to 0.008)	0.310
PR interval	ms	0.047 (-0.003 to 0.005)	0.656
QRS duration	ms	0.351 (0.004 to 0.014)	<0.001
QT interval	ms	0.237 (0.000 to 0.004)	0.018
QTc interval	ms	0.336 (0.002–0.006)	0.001

HDL: high-density lipoprotein, LDL: low-density lipoprotein.

### Patient clinical laboratory data stratified by QTc status

The patients were divided into the normal QTc and prolonged QTc groups according to the criteria of AHA/ACCF/HRS [22] to investigate whether biological factors affect QTc (Table 3). Of the 100 patients in our study, 26 patients had prolonged QTc and had significantly higher serum IS levels but lower HDL levels compared to those of the normal QTc patients even after adjusting for age and sex.

**Table 3:** Patient clinical laboratory data according to QTc classification.

	QTc <450 ms in women / <460 ms in men	QTc ≥450 ms in women / ≥460 ms in men	p-value
No	74	26	
Age (years)	59.0 ± 9.0	66.4 ± 11.4	0.002
Sex (male/female)	44/30	12/14	0.260
Current smoking (n, %)	23 (31.1)	9 (34.6)	0.805
BMI (kg/m^2^)	25.0 ± 3.2	25.7 ± 4.3	0.437
Systolic BP (mmHg)	128 ± 14	130 ± 31	0.620
Diastolic BP (mmHg)	76 ± 10	73 ± 13	0.341
Fasting glucose (mg/dl)	105.8 ± 23.9	126.8 ± 59.4	0.300
Total cholesterol (mg/dl)	184.3 ± 36.5	170.8 ± 54.0	0.071
Triglyceride (mg/dl)	103.5 (74.5–173.5)	112.0 (81.8–176.5)	0.506
HDL-cholesterol (mg/dl)	46.1 ± 12.1	40.5 ± 14.6	0.007
LDL-cholesterol (mg/dl)	107.6 ± 31.5	101.3 ± 51.8	0.216
NA (mEq/L)	139.9 ± 2.5	139.0 ± 4.0	0.190
K (mEq/L)	4.0 ± 0.8	4.0 ± 0.6	0.682
Calcium (mg/dl)	8.5 ± 1.1	8.8 ± 0.7	0.184
Hematocrit (%)	40.6 ± 4.6	39.5 ± 4.5	0.293
Creatinine (mg/dl)	1.1 (1.0–1.2)	1.1 (1.0–1.7)	0.157
Albumin (mg/dl)	4.1 ± 0.3	4.0 ± 0.4	0.218
Estimated GFR (ml/min/1.73m^2^)	64.6 ± 12.2	57.8 ± 20.1	0.343
Indoxyl sulfate (μmol/L)	2.8 (0.9–5.2)	6.1 (0.9–11.3)	0.019

Data are expressed as mean ± SD, number (%), or median (interquartile range). BMI, body mass index; BP, blood pressure; HDL, high-density lipoprotein; LDL, low-density lipoprotein.

### Association between IS and prolonged QTc interval

Multivariate logistic regression analysis was performed to estimate the effects of serum IS level together with several other parameters in the presence of prolonged QTc interval. The presence of prolonged QTc interval was associated with age, sex, and serum IS level (Table 4).

**Table 4:** Multiple logistic regression analysis with presence of prolonged QTc interval as the dependent variable.

	exp(B)	95% Confidence Interval	p-value
Age	4.94	1.04–2.44	0.045
Sex	0.05	0.01–0.59	0.017
BMI	1.12	0.91–1.38	0.285
Systolic BP	1.02	0.98–1.06	0.424
Diastolic BP	0.97	0.91–1.05	0.458
Fasting glucose	1.02	0.99–1.04	0.087
Total cholesterol	1.02	0.95–1.09	0.547
Triglyceride	0.99	0.98–1.01	0.312
HDL-cholesterol	0.95	0.86–1.04	0.270
LDL-cholesterol	0.98	0.92–1.05	0.548
Smoking	9.19	0.91–10.16	0.061
Na	0.89	0.66–1.19	0.428
K	0.55	0.18–1.66	0.285
Calcium	1.50	0.63–3.56	0.358
Indoxyl sulfate	7.35	1.12–4.48	0.037

BMI, body mass index; BP, blood pressure; HDL, high-density lipoprotein; LDL, low-density lipoprotein.

### The effect of IS on H9c2 ventricular cardiomyocyte potassium outward current

To evaluate the effect of IS on H9c2 cell, in the beginning, we tested the acute effect of IS on the delayed rectifier potassium current in cardiac H9c2 cells with an IS treatment of 16 hours over night. However, there was no significant change in the potassium current in the treated group compared to that of the controls. As a result, we prolong the duration of treatment to 48 hours and founded that the potassium current was significantly decreased in the IS treated group compared to that of the controls. To our knowledge, there are few reports investigating IS effect on cardiomyocytes, though Lekawanvijit et al. found that IS induced cardiomyocyte hypertrophic change after 48 hours of IS treatment [24]. For this reason, we designed the condition of cell incubation for 48 hours.

**Fig 1:**
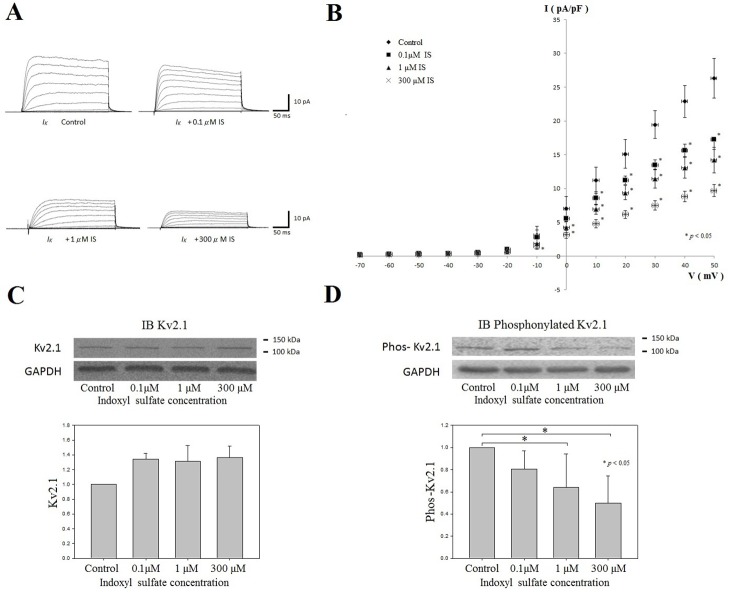
The effect of IS on H9c2 cardiomyocyte *I*_*K*_ and potassium channel protein Kv2.1 expression. (A) The representative current traces for delayed rectifier potassium outward currents (*I*_***k***_) in H9c2 cells with different indoxyl sulfate (IS) concentration treatment. *I*_***k***_ were elicited by 300 ms depolarizing step pulses from -70 to 50 mV at a holding potential of -60 mV. (B) The average relationships between *I*_***k***_ (pA/pF) and membrane potential in the control, 0.1μM IS, 1 μM IS and 300μM IS groups (n = 6 for each groups) comparing the IS treated group with the control group, *I*_***k***_ was significantly decreased at membrane potentials from 0 mV to 50 mV in a dose-dependent manner. (C and D) The expression of Kv2.1 and phosphorylated Kv2.1 by Western blot in the H9c2 cells treated with different concentration of IS (0.1μM, 1 μM and 300μM). The expressions of Kv2.1 were not significantly different among the control and IS-treated groups (C). However, the phosphorylated Kv2.1 was significantly decreased in the 1 μM IS-and 300 μM-IS treated groups (D). (n = 6 for each groups) *: *p*<0.05 as compared with the control group.

The results of the patch-clamp cell electrophysiological study revealed that the *I*_*K*_ was significantly decreased after treatment with IS for 48 hours (Fig 1A). The average relationships between *I*_*K*_ and membrane potential calculated from the measured peak current amplitudes showed that *I*_*K*_ was significantly decreased at the membrane potentials from 0 mV to 50 mV in a dose-dependent manner (Fig 1B).

### Kv2.1 Western Blot Analysis with and without IS treatment in H9c2 cardiomyocyte

Potassium ion channel protein Kv2.1 is the major subunit protein comprised of ion channels that generate *I*_*K*_ in H9c2 cell [23]. Western Blot analysis revealed that there was an increase in Kv2.1 level after the treatment of IS. However, there were no significant differences noted between the groups (Fig 1C). In contrast, the expression of phosphorylated Kv2.1 was found to be significantly decreased in the IS-treated groups (Fig 1D).

### Mathematical computer simulation of IS effect on human cardiomyocyte electrophysiology

**Fig 2:**
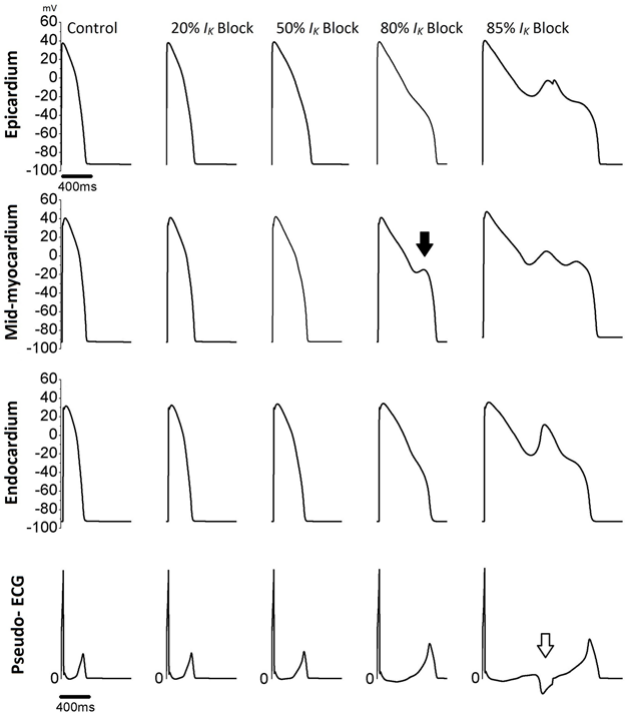
Ventricular cardiomyocyte action potential (AP) and pseudo-ECG constructed by the O'Hara-Rudy dynamic human ventricular model. The suppression of inward rectifier potassium current (*I*_***K***_) mimics the effect of indoxyl sulfate toxicity to ventricular cardiomyocyte AP. The AP duration was gradually increased and the QT interval was also prolonged with the increment of *I*_***K***_ suppression. The early afterdepolarization was noted in the higher suppression of *I*_***K***_ especially in the mid-myocardial cardiomyocyte (arrow). The ventricular arrhythmias like ECG was also noted when the *I*_***K***_ was severely suppressed (empty arrow).

From the computer calculations and simulation results, the decrease of *I*_*K*_ caused by the increase of treated IS concentration will gradually prolong the constructed action potential duration (APD) and pseudo ECG QT interval (Fig 2). In addition, early afterdepolarization (EAD) was noted in the higher suppression of *I*_*K*_ simulation, which mimicked the high IS effect on the cell electrophysiological studies (Fig 2, arrow). The constructed pseudo ECG also showed the ventricular arrhythmias like ECG when the *I*_*K*_ was severely suppressed (Fig 2, empty arrow).

## Discussion

In the present study, we demonstrated that serum IS concentrations are correlated with age, hematocrit, creatinine, estimated GFR, albumin, QRS duration, and QTc interval. The serum IS concentration was increased in QTc prolongation patients in contrast with the normal QTc controls; in addition, multiple logistic regression analysis also confirmed this independent association, even in a fully adjusted model. Furthermore, in cell electrophysiological study, IS decreased the *I*_*K*_ in rat ventricular cardiomyocyte through the regulation of the major potassium ion channel protein Kv 2.1 phosphorylation. To our knowledge, this is the first report to observe that the serum IS level is associated with QTc prolongation and that the possible mechanism of this phenomenon is through the down-regulation of *I*_*K*_ channel protein phosphorylation and the *I*_*K*_ current activity that in turn increases the APD and QTc interval.

QT prolongation is usually found in patients with chronic renal diseases and multiple mechanisms have been suggested, such as electrolyte imbalance, autonomic nerve dysfunction, rapid changes in electrolyte plasma concentrations during hemodialysis and cardiac hypertrophy [34]. However, there have been few reports demonstrating the relationship between early stages of CKD and QT interval. Cardiorenal syndrome (CRS), which indicates that in the heart and kidney, acute or chronic dysfunction of one organ may induce acute or chronic dysfunction in the other, is caused by multiple factors, including non-dialyzable uremic toxins, such as IS [35].

Recently, IS has been reported to have the profibrotic and prohypertropic effects on cardiomyocytes [24], free radical production [7], endothelial microparticle release [36], vascular smooth muscle cell proliferation [37], and adherens junctions disruption of vascular endothelial cells [38]. Clinically, all of these pathogenetic states can contribute to vascular damage [39], progression of renal diseases [40], coronary artery disease [10], and even mortality [39] in CKD patients. In the present study, we found that there is a strong association between QTc interval and serum IS level in early CKD patients. Importantly, an increase in circulating IS levels can occur in the early stages (2 and 3) of CKD [41], and the combined prevalence of stages 2 and 3 accounts for 67% of all CKD stages [42].

The biological mechanisms involving IS level in the pathogenesis of QTc prolongation is not well understood. According to Ronco et al., IS is the strongest evidence-based uremic toxin involved in type 2 and type 4 CRS pathophysiology, which is mainly attributable to the profibrotic effects [35]. Recently, IS has also been proven to have a direct effect on cardiac fibroblasts and induced cardiac fibrosis in an animal study [24]. As cardiac fibrosis is known as one of the mechanisms of cardiac dysfunction, QT prolongation and cardiac arrhythmia [43], as well as high serum IS levels may be associated with QTc prolongation. In addition, disturbances in gap junctional intercellular communication affect the electrical coupling between heart muscle cells and the underlie prolongation of QRS and QT intervals [44]. Several uremic retention toxins, such as homocysteine [45] and *p*-cresol [46] have been shown to be involved in the disassembly of connexin and the disruption of the adherens junction of cardiomyocyte and QT prolongation in an animal model [45]. Therefore, whether IS also involved in the modulation of cardiomyocyte connexin needs further investigation.

*I*_*K*_ is one of the core current determinants of the cardiomyocyte APD, with the increase of APD prolonging the ECG QT interval [13]. The Kv2.1 protein is the major subunit protein comprising the ion channel in H9c2 cell, which generates the *I*_*K*_ [23], and previous studies have also shown the Kv2.1 channel activity is regulated by phosphorylation [47]. Here, we demonstrated that IS decreased the expression of phosphorylated Kv2.1 in the H9c2 cell, down-regulated the Kv2.1 channel activity and decreased *I*_*K*_, which suggests that IS may play a role in the development of QTc prolongation. This finding provides evidence that uremic toxins are related to arrhythmogenesis, and beyond the present traditional risk factors. From a literature review, we uncovered no documented mechanism describing IS as down-regulates phopho-Kv2.1. However, Park et al. showed that the Kv2.1 potassium channel is regulated by variable phosphorylation [48], in which protein kinase C (PKC) is usually involved [47,49]. Previous studies have demonstrated that PKC was associated with inflammation, oxidative stress, and many other pathological states [50,51]. IS has also been proven to be involved with free radical production and the elevation of inflammatory mediators [6,7,9]. In addition, the induction of nephrotoxicity by IS is mediated by organic anion transporters (OATs), such as OAT1 and OAT3, which are localized in the basolateral membrane of renal proximal tubular cells [52]; OAT1 activity/function is modulated via phosphorylation mediated by the PKC pathway [53]. Hence, it is possible that IS might down regulates phopho-Kv2.1 via the PKC pathway, but further experiments and studies will need to verify this.

It is hard to demonstrate any channelopathies in human cardiomyocyte and their consequent effects on the human heart surface ECG. Recently, both experimental and theoretical models have been used in procedures studying the biological factors that induce arrhythmias [30]. In our computer simulation experiment, the prolonged APD, EAD phenomenon, prolonged QT interval and ventricular arrhythmias-like ECG appearance were noted (Fig 2). The EAD induction and trigger activity were the major arrhythmogenesis in ventricular arrhythmias. The induction of EAD in the experiment suggests an IS arrhythmogenic effect and its possible role in the arrhythmias and SCD among CKD patients.

Among these non-dialyzable uremic toxins, many biological and pathological effects have been previously discussed [54,55]. However, there are lacks of reports showing the arrhythmogenic effect of these non-dailyzable uremic toxins in human heart or animal models. IS is the representative molecule of uremic toxin and already known to be associated with the pathogenesis of many uremic syndrome. Previous studies have shown that IS has profibrotic and prohypertropic effects on cardiomyocytes [24,56], which are also known to be related to free radical production and the elevation of inflammatory mediators, which are in turn proven to affect cardiac ion channel function [24,48,49,56]. It is therefore reasonable to select IS over other toxins for investigation into its arrhythmogenic effect. Further work is required to confirm these findings in other uremic toxins.

Some limitations of this study need to be considered. First, our study population was relatively small. Further, the cross-sectional design limits our ability to infer any causal relationship between total IS levels and QTc prolongation. Second, we did not determined the plasma magnesium and bicarbonate levels in our patients due to hospital standard cardiac multi-slice CT or coronary angiography examination preparation protocols; as such, it is unfortunate that we could not demonstrate whether these electrolytes also affect QTc prolongation. Third, in our study, the IS affected *I*_*K*_ in vitro at very low concentrations, even below the normal population serum IS level [57]. Moreover, the potassium concentration in the added IS potassium salt (0.1uM to 0.3mM) was relatively low compared to the potassium concentration in the DMEM culture medium (5.3mM) used in the experiment. Hence, the additional potassium effect on the action potentials in the experiment could have been eliminated. As the cardiac electrical activity is a complex system, it is no doubt plausible that there exists another modulation system in vivo to contend with the IS biological effect, such as the equilibrium state of IS protein binding capacity [24]. Finally, it is still unclear whether IS affects the expression of other cardiac ion channels, ion currents and cardiomyocyte electrophysiology. Further investigation is warranted.

In conclusion, our study demonstrated that the QTc interval was prolonged in early CKD patients with a higher serum IS level. The arrhythmogenic effect of IS was shown through the inhibition of *I*_*K*_. By the progress of renal disease and the interaction of CRS, the effect and role of IS on the arrhythmogenesis among CKD patients might be enhanced in conjunction with the advance of renal function impairment. As a result, the arrhythmogenic effect of IS should be taken seriously.

## Supporting Information

S1 Code Mathematical computer model for cardiomyocyte action potential and pseudo-ECG. In our experiment, cardiomyocyte action potential was mathematically constructed by the latest mathematical model of the O’Hara-Rudy dynamic human ventricular model. The codes and equations we used in our experiment were downloaded and modified from the Open access and supplemental material journal PLoS computational Biology.
(DOC)
