# Supplementary material for: Uremic Retention Solute Indoxyl Sulfate Level Is Associated with Prolonged QTc Interval in Early CKD Patients
Source: PLoS One. 2015 Apr 20;10(4):e0119545. doi: 10.1371/journal.pone.0119545 (PMC4403985; doi:10.1371/journal.pone.0119545)
Supplement: S1 Code — In our experiment, cardiomyocyte action potential was mathematically constructed by the latest mathematical model of the O’Hara-Rudy dynamic human ventricular model. The codes and equations we used in our experiment were downloaded and modified from the Open access and supplemental material journal PLoS computational Biology. (DOC) [file pone.0119545.s001.doc]

**S1 Mathematical computer model for cardiomyocyte action potential and pseudo-ECG**

In our experiment, cardiomyocyte action potential was mathematically constructed by the latest mathematical model of O’Hara-Rudy dynamic human ventricular model. The following is the codes and equations we had used in our experiment. It was downloaded from the Open access and supplemental material journal PLoS computational Biology and could also be downloaded freely from the website: <http://rudylab.wustl.edu/research/cell/code/AllCodes.html> [ 1 ].

#include <math.h>

#include <iostream>

using namespace std;

#include <fstream>

using std::ifstream;

void revpots();//compute reversal potentials

void RGC();//compute rates, gates, and currents

void stimulus();//determine the value for the periodic stimulus

void voltage();//calculate teh new membrane voltage

void dVdt_APD();//caluculate voltage derivative and APD90

void FBC();//calculate fluxes, buffers, and concentrations

const double CL=1000;//pacing cycle length

const double ft=1000*CL;//final time

const int skip=10;//number of timesetps to skip in sampling of data in output file

const double safetime=25.0;//time from the beginning of each beat during which dt is fixed to small values

const double beatssave=2;//number of beats to save in the output

const double amp=-80;//stimulus amplitude in uA/uF

const double start=0;//start time of the stimulus, relative to each beat

const double duration=0.5;//duration of teh stimulus in ms

const int celltype=0; //endo = 0, epi = 1, M = 2

//initial values for state variables, there are 41 of them

double v=-87.5;

double nai=7;

double nass=nai;

double ki=145;

double kss=ki;

double cai=1.0e-4;

double cass=cai;

double cansr=1.2;

double cajsr=cansr;

double m=0;

double hf=1;

double hs=1;

double j=1;

double hsp=1;

double jp=1;

double mL=0;

double hL=1;

double hLp=1;

double a=0;

double iF=1;

double iS=1;

double ap=0;

double iFp=1;

double iSp=1;

double d=0;

double ff=1;

double fs=1;

double fcaf=1;

double fcas=1;

double jca=1;

double nca=0;

double ffp=1;

double fcafp=1;

double xrf=0;

double xrs=0;

double xs1=0;

double xs2=0;

double xk1=1;

double Jrelnp=0;

double Jrelp=0;

double CaMKt=0;

//constants

double const nao=140.0;//extracellular sodium in mM

double const cao=1.8;//extracellular calcium in mM

double const ko=5.4;//extracellular potassium in mM

//buffer paramaters

double const BSRmax=0.047;

double const KmBSR=0.00087;

double const BSLmax=1.124;

double const KmBSL=0.0087;

double const cmdnmax=0.05;

double const kmcmdn=0.00238;

double const trpnmax=0.07;

double const kmtrpn=0.0005;

double const csqnmax=10.0;

double const kmcsqn=0.8;

//CaMK paramaters

double const aCaMK=0.05;

double const bCaMK=0.00068;

double const CaMKo=0.05;

double const KmCaM=0.0015;

double const KmCaMK=0.15;

//physical constants

double const R=8314.0;

double const T=310.0;

double const F=96485.0;

//cell geometry

double const L=0.01;

double const rad=0.0011;

double const vcell=1000*3.14*rad*rad*L;

double const Ageo=2*3.14*rad*rad+2*3.14*rad*L;

double const Acap=2*Ageo;

double const vmyo=0.68*vcell;

double const vmito=0.26*vcell;

double const vsr=0.06*vcell;

double const vnsr=0.0552*vcell;

double const vjsr=0.0048*vcell;

double const vss=0.02*vcell;

//introduce varaibles for reversal potentials, currents, fluxes, and CaMK

double ENa,EK,EKs;

double INa,INaL,Ito,ICaL,ICaNa,ICaK,IKr,IKs,IK1,INaCa_i,INaCa_ss,INaCa,INaK,IKb,INab,IpCa,ICab,Ist;

double Jrel,Jup,Jtr,Jdiff,JdiffNa,JdiffK,Jleak;

double CaMKa,CaMKb;

//introduce APD, timing, and counting parameters

int APD_flag=0;

double APD;

double t_vdot_max;

double vrest;

double vo=v;

double dt=0.005;

double t0=0;

double t=0;

double dto;

double vdot_old;

double vdot=0;

double vdot_max;

int p=1;

int n=0;

int count=1;

//value holders for state varaibles in the case that the increase in dt was too aggressive, so a smaller one can be taken

double nai0,nass0,ki0,kss0,cai0,cass0,cansr0,cajsr0,m0,hf0,hs0,jO,hsp0,jp0,mL0,hL0,hLp0,a0,iF0,iS0,ap0,iFp0,iSp0,d0,ff0,fs0,fcaf0,fcas0,jca0,nca0,ffp0,fcafp0,xrf0,xrs0,xs10,xs20,xk10,Jrelnp0,Jrelp0,CaMKt0;

int main()

{

//establish the output file "output.txt"

FILE*output;

output = fopen("output.txt","w");

fprintf(output,"%-07s\t%-07s\t%-07s\t%-07s\t%-07s\t%-07s\t%-07s\t%-07s\t%-07s\t%-07s\t%-07s\t%-07s\t%-07s\t%-07s\t%-07s\t%-07s\t%-07s\t%-07s\t%-07s\t%-07s\t%-07s\t%-07s\t%-07s\t%-07s\t%-07s\t%-07s\t%-07s\t%-07s\t%-07s\t%-07s\t%-07s\t%-07s\t%-07s\t%-07s\t%-07s\t%-07s\t%-07s\t%-07s\t%-07s\n",

"variables=","t","v","nai","nass","ki","kss","cai","cass","cansr","cajsr","Jrel","CaMKt","Jup","Jtr","Jdiff","JdiffNa","JdiffK","Jleak","INa","INaL","Ito","ICaL","ICaNa","ICaK","IKr","IKs","IK1","INaCa_i","INaCa_ss","INaCa","INaK","IKb","INab","IpCa","ICab","Ist","dt","APD");

fclose(output);

output = fopen("output.txt","a");//open the output file for appending new data

while (t<=ft)

{

//rules for dynamic dt choice, and model integration, comment to use fixed time steps

if ((t>=(start+n*CL-2) && t<(start+duration+n*CL)) || (n>=1 && t<(start+duration+(n-1)*CL+safetime)) || (APD_flag==1 && v<0.7*vrest))

{

dt=0.005;

t=t+dt;

revpots();

RGC();

stimulus();

vo=v;

voltage();

dVdt_APD();

FBC();

}

else if (fabs(v-vo)<0.2)

{

dt=fabs(0.8/vdot);

if (dt>1.0)

{

dt=1.0;

}

t=t+dt;

revpots();

RGC();

stimulus();

vo=v;

voltage();

dVdt_APD();

FBC();

}

else if (fabs(v-vo)>0.8)

{

nai0=nai;

nass0=nass;

ki0=ki;

cai0=cai;

cass0=cass;

cansr0=cansr;

cajsr0=cajsr;

m0=m;

hf0=hf;

hs0=hs;

jO=j;

hsp0=hsp;

jp0=jp;

mL0=mL;

hL0=hL;

hLp0=hLp;

a0=a;

iF0=iF;

iS0=iS;

ap0=ap;

iFp0=iFp;

iSp0=iSp;

d0=d;

ff0=ff;

fs0=fs;

fcaf0=fcaf;

fcas0=fcas;

jca0=jca;

nca0=nca;

ffp0=ffp;

fcafp=fcafp;

xrf0=xrf;

xrs0=xrs;

xs10=xs1;

xs20=xs2;

xk10=xk1;

Jrelnp0=Jrelnp;

Jrelp0=Jrelp;

CaMKt0=CaMKt;

t0=t;

dto=dt;

dt=fabs(0.2/vdot);

t=t+dt;

revpots();

RGC();

stimulus();

vo=v;

voltage();

dVdt_APD();

FBC();

while (fabs(v-vo)>0.8)

{

v=vo;

nai=nai0;

nass=nass0;

ki=ki0;

cai=cai0;

cass=cass0;

cansr=cansr0;

cajsr=cajsr0;

m=m0;

hf=hf0;

hs=hs0;

j=jO;

hsp=hsp0;

jp=jp0;

mL=mL0;

hL=hL0;

hLp=hLp0;

a=a0;

iF=iF0;

iS=iS0;

ap=ap0;

iFp=iFp0;

iSp=iSp0;

d=d0;

ff=ff0;

fs=fs0;

fcaf=fcaf0;

fcas=fcas0;

jca=jca0;

nca=nca0;

ffp=ffp0;

fcafp=fcafp0;

xrf=xrf0;

xrs=xrs0;

xs1=xs10;

xs2=xs20;

xk1=xk10;

Jrelnp=Jrelnp0;

Jrelp=Jrelp0;

CaMKt=CaMKt0;

if (p==1)

{

dt=dto-0.01;

p=0;

}

else

{

dt=dt-0.01;

}

if (dt<=0)

{

dt=1e-6;

}

t=t0+dt;

revpots();

RGC();

stimulus();

voltage();

dVdt_APD();

FBC();

}

p=1;

}

else

{

t=t+dt;

revpots();

RGC();

stimulus();

vo=v;

voltage();

dVdt_APD();

FBC();

}

//uncomment below, and comment above to use a fixed dt

/*t=t+dt; //fixed time step

revpots();

RGC();

stimulus();

vo=v;

voltage();

dVdt_APD();

FBC();*/

if (count%500000==0)

{

cout<<t/ft*100<<"% complete"<<endl;//output runtime progress to the screen

}

if (count%skip==0 && t>=ft-beatssave*CL)//save results ot output file when the sampling interval and time are correct

{

fprintf(output,"%-018e\t%-07g\t%-07g\t%-07g\t%-07g\t%-07g\t%-07g\t%-07g\t%-07g\t%-07g\t%-07g\t%-07g\t%-07g\t%-07g\t%-07g\t%-07g\t%-07g\t%-07g\t%-07g\t%-07g\t%-07g\t%-07g\t%-07g\t%-07g\t%-07g\t%-07g\t%-07g\t%-07g\t%-07g\t%-07g\t%-07g\t%-07g\t%-07g\t%-07g\t%-07g\t%-07g\t%-07g\t%-07g\n",

t-(ft-beatssave*CL),v,nai,nass,ki,kss,cai,cass,cansr,cajsr,Jrel,CaMKt,Jup,Jtr,Jdiff,JdiffNa,JdiffK,Jleak,INa,INaL,Ito,ICaL,ICaNa,ICaK,IKr,IKs,IK1,INaCa_i,INaCa_ss,INaCa,INaK,IKb,INab,IpCa,ICab,Ist,dt,APD);

}

count++;//increase the loop counter

}

fclose(output);//close the output file

return 0;

}

void revpots()

{

ENa=(R*T/F)*log(nao/nai);

EK=(R*T/F)*log(ko/ki);

EKs=(R*T/F)*log((ko+0.01833*nao)/(ki+0.01833*nai));

}

void RGC()

{

CaMKb=CaMKo*(1.0-CaMKt)/(1.0+KmCaM/cass);

CaMKa=CaMKb+CaMKt;

double vffrt=v*F*F/(R*T);

double vfrt=v*F/(R*T);

double mss=1.0/(1.0+exp((-(v+39.57))/9.871));

double tm=1.0/(6.765*exp((v+11.64)/34.77)+8.552*exp(-(v+77.42)/5.955));

m=mss-(mss-m)*exp(-dt/tm);

double hss=1.0/(1+exp((v+82.90)/6.086));

double thf=1.0/(1.432e-5*exp(-(v+1.196)/6.285)+6.149*exp((v+0.5096)/20.27));

double ths=1.0/(0.009794*exp(-(v+17.95)/28.05)+0.3343*exp((v+5.730)/56.66));

double Ahf=0.99;

double Ahs=1.0-Ahf;

hf=hss-(hss-hf)*exp(-dt/thf);

hs=hss-(hss-hs)*exp(-dt/ths);

double h=Ahf*hf+Ahs*hs;

double jss=hss;

double tj=2.038+1.0/(0.02136*exp(-(v+100.6)/8.281)+0.3052*exp((v+0.9941)/38.45));

j=jss-(jss-j)*exp(-dt/tj);

double hssp=1.0/(1+exp((v+89.1)/6.086));

double thsp=3.0*ths;

hsp=hssp-(hssp-hsp)*exp(-dt/thsp);

double hp=Ahf*hf+Ahs*hsp;

double tjp=1.46*tj;

jp=jss-(jss-jp)*exp(-dt/tjp);

double GNa=75;

double fINap=(1.0/(1.0+KmCaMK/CaMKa));

INa=GNa*(v-ENa)*m*m*m*((1.0-fINap)*h*j+fINap*hp*jp);

double mLss=1.0/(1.0+exp((-(v+42.85))/5.264));

double tmL=tm;

mL=mLss-(mLss-mL)*exp(-dt/tmL);

double hLss=1.0/(1.0+exp((v+87.61)/7.488));

double thL=200.0;

hL=hLss-(hLss-hL)*exp(-dt/thL);

double hLssp=1.0/(1.0+exp((v+93.81)/7.488));

double thLp=3.0*thL;

hLp=hLssp-(hLssp-hLp)*exp(-dt/thLp);

double GNaL=0.0075;

if (celltype==1)

{

GNaL*=0.6;

}

double fINaLp=(1.0/(1.0+KmCaMK/CaMKa));

INaL=GNaL*(v-ENa)*mL*((1.0-fINaLp)*hL+fINaLp*hLp);

double ass=1.0/(1.0+exp((-(v-14.34))/14.82));

double ta=1.0515/(1.0/(1.2089*(1.0+exp(-(v-18.4099)/29.3814)))+3.5/(1.0+exp((v+100.0)/29.3814)));

a=ass-(ass-a)*exp(-dt/ta);

double iss=1.0/(1.0+exp((v+43.94)/5.711));

double delta_epi;

if (celltype==1)

{

delta_epi=1.0-(0.95/(1.0+exp((v+70.0)/5.0)));

}

else

{

delta_epi=1.0;

}

double tiF=4.562+1/(0.3933*exp((-(v+100.0))/100.0)+0.08004*exp((v+50.0)/16.59));

double tiS=23.62+1/(0.001416*exp((-(v+96.52))/59.05)+1.780e-8*exp((v+114.1)/8.079));

tiF*=delta_epi;

tiS*=delta_epi;

double AiF=1.0/(1.0+exp((v-213.6)/151.2));

double AiS=1.0-AiF;

iF=iss-(iss-iF)*exp(-dt/tiF);

iS=iss-(iss-iS)*exp(-dt/tiS);

double i=AiF*iF+AiS*iS;

double assp=1.0/(1.0+exp((-(v-24.34))/14.82));

ap=assp-(assp-ap)*exp(-dt/ta);

double dti_develop=1.354+1.0e-4/(exp((v-167.4)/15.89)+exp(-(v-12.23)/0.2154));

double dti_recover=1.0-0.5/(1.0+exp((v+70.0)/20.0));

double tiFp=dti_develop*dti_recover*tiF;

double tiSp=dti_develop*dti_recover*tiS;

iFp=iss-(iss-iFp)*exp(-dt/tiFp);

iSp=iss-(iss-iSp)*exp(-dt/tiSp);

double ip=AiF*iFp+AiS*iSp;

double Gto=0.02;

if (celltype==1)

{

Gto*=4.0;

}

if (celltype==2)

{

Gto*=4.0;

}

double fItop=(1.0/(1.0+KmCaMK/CaMKa));

Ito=Gto*(v-EK)*((1.0-fItop)*a*i+fItop*ap*ip);

double dss=1.0/(1.0+exp((-(v+3.940))/4.230));

double td=0.6+1.0/(exp(-0.05*(v+6.0))+exp(0.09*(v+14.0)));

d=dss-(dss-d)*exp(-dt/td);

double fss=1.0/(1.0+exp((v+19.58)/3.696));

double tff=7.0+1.0/(0.0045*exp(-(v+20.0)/10.0)+0.0045*exp((v+20.0)/10.0));

double tfs=1000.0+1.0/(0.000035*exp(-(v+5.0)/4.0)+0.000035*exp((v+5.0)/6.0));

double Aff=0.6;

double Afs=1.0-Aff;

ff=fss-(fss-ff)*exp(-dt/tff);

fs=fss-(fss-fs)*exp(-dt/tfs);

double f=Aff*ff+Afs*fs;

double fcass=fss;

double tfcaf=7.0+1.0/(0.04*exp(-(v-4.0)/7.0)+0.04*exp((v-4.0)/7.0));

double tfcas=100.0+1.0/(0.00012*exp(-v/3.0)+0.00012*exp(v/7.0));

double Afcaf=0.3+0.6/(1.0+exp((v-10.0)/10.0));

double Afcas=1.0-Afcaf;

fcaf=fcass-(fcass-fcaf)*exp(-dt/tfcaf);

fcas=fcass-(fcass-fcas)*exp(-dt/tfcas);

double fca=Afcaf*fcaf+Afcas*fcas;

double tjca=75.0;

jca=fcass-(fcass-jca)*exp(-dt/tjca);

double tffp=2.5*tff;

ffp=fss-(fss-ffp)*exp(-dt/tffp);

double fp=Aff*ffp+Afs*fs;

double tfcafp=2.5*tfcaf;

fcafp=fcass-(fcass-fcafp)*exp(-dt/tfcafp);

double fcap=Afcaf*fcafp+Afcas*fcas;

double Kmn=0.002;

double k2n=1000.0;

double km2n=jca*1.0;

double anca=1.0/(k2n/km2n+pow(1.0+Kmn/cass,4.0));

nca=anca*k2n/km2n-(anca*k2n/km2n-nca)*exp(-km2n*dt);

double PhiCaL=4.0*vffrt*(cass*exp(2.0*vfrt)-0.341*cao)/(exp(2.0*vfrt)-1.0);

double PhiCaNa=1.0*vffrt*(0.75*nass*exp(1.0*vfrt)-0.75*nao)/(exp(1.0*vfrt)-1.0);

double PhiCaK=1.0*vffrt*(0.75*kss*exp(1.0*vfrt)-0.75*ko)/(exp(1.0*vfrt)-1.0);

double zca=2.0;

double PCa=0.0001;

if (celltype==1)

{

PCa*=1.2;

}

if (celltype==2)

{

PCa*=2.5;

}

double PCap=1.1*PCa;

double PCaNa=0.00125*PCa;

double PCaK=3.574e-4*PCa;

double PCaNap=0.00125*PCap;

double PCaKp=3.574e-4*PCap;

double fICaLp=(1.0/(1.0+KmCaMK/CaMKa));

ICaL=(1.0-fICaLp)*PCa*PhiCaL*d*(f*(1.0-nca)+jca*fca*nca)+fICaLp*PCap*PhiCaL*d*(fp*(1.0-nca)+jca*fcap*nca);

ICaNa=(1.0-fICaLp)*PCaNa*PhiCaNa*d*(f*(1.0-nca)+jca*fca*nca)+fICaLp*PCaNap*PhiCaNa*d*(fp*(1.0-nca)+jca*fcap*nca);

ICaK=(1.0-fICaLp)*PCaK*PhiCaK*d*(f*(1.0-nca)+jca*fca*nca)+fICaLp*PCaKp*PhiCaK*d*(fp*(1.0-nca)+jca*fcap*nca);

double xrss=1.0/(1.0+exp((-(v+8.337))/6.789));

double txrf=12.98+1.0/(0.3652*exp((v-31.66)/3.869)+4.123e-5*exp((-(v-47.78))/20.38));

double txrs=1.865+1.0/(0.06629*exp((v-34.70)/7.355)+1.128e-5*exp((-(v-29.74))/25.94));

double Axrf=1.0/(1.0+exp((v+54.81)/38.21));

double Axrs=1.0-Axrf;

xrf=xrss-(xrss-xrf)*exp(-dt/txrf);

xrs=xrss-(xrss-xrs)*exp(-dt/txrs);

double xr=Axrf*xrf+Axrs*xrs;

double rkr=1.0/(1.0+exp((v+55.0)/75.0))*1.0/(1.0+exp((v-10.0)/30.0));

double GKr=0.046;

if (celltype==1)

{

GKr*=1.3;

}

if (celltype==2)

{

GKr*=0.8;

}

IKr=GKr*sqrt(ko/5.4)*xr*rkr*(v-EK);

double xs1ss=1.0/(1.0+exp((-(v+11.60))/8.932));

double txs1=817.3+1.0/(2.326e-4*exp((v+48.28)/17.80)+0.001292*exp((-(v+210.0))/230.0));

xs1=xs1ss-(xs1ss-xs1)*exp(-dt/txs1);

double xs2ss=xs1ss;

double txs2=1.0/(0.01*exp((v-50.0)/20.0)+0.0193*exp((-(v+66.54))/31.0));

xs2=xs2ss-(xs2ss-xs2)*exp(-dt/txs2);

double KsCa=1.0+0.6/(1.0+pow(3.8e-5/cai,1.4));

double GKs=0.0034;

if (celltype==1)

{

GKs*=1.4;

}

IKs=GKs*KsCa*xs1*xs2*(v-EKs);

double xk1ss=1.0/(1.0+exp(-(v+2.5538*ko+144.59)/(1.5692*ko+3.8115)));

double txk1=122.2/(exp((-(v+127.2))/20.36)+exp((v+236.8)/69.33));

xk1=xk1ss-(xk1ss-xk1)*exp(-dt/txk1);

double rk1=1.0/(1.0+exp((v+105.8-2.6*ko)/9.493));

double GK1=0.1908;

if (celltype==1)

{

GK1*=1.2;

}

if (celltype==2)

{

GK1*=1.3;

}

IK1=GK1*sqrt(ko)*rk1*xk1*(v-EK);

double kna1=15.0;

double kna2=5.0;

double kna3=88.12;

double kasymm=12.5;

double wna=6.0e4;

double wca=6.0e4;

double wnaca=5.0e3;

double kcaon=1.5e6;

double kcaoff=5.0e3;

double qna=0.5224;

double qca=0.1670;

double hca=exp((qca*v*F)/(R*T));

double hna=exp((qna*v*F)/(R*T));

double h1=1+nai/kna3*(1+hna);

double h2=(nai*hna)/(kna3*h1);

double h3=1.0/h1;

double h4=1.0+nai/kna1*(1+nai/kna2);

double h5=nai*nai/(h4*kna1*kna2);

double h6=1.0/h4;

double h7=1.0+nao/kna3*(1.0+1.0/hna);

double h8=nao/(kna3*hna*h7);

double h9=1.0/h7;

double h10=kasymm+1.0+nao/kna1*(1.0+nao/kna2);

double h11=nao*nao/(h10*kna1*kna2);

double h12=1.0/h10;

double k1=h12*cao*kcaon;

double k2=kcaoff;

double k3p=h9*wca;

double k3pp=h8*wnaca;

double k3=k3p+k3pp;

double k4p=h3*wca/hca;

double k4pp=h2*wnaca;

double k4=k4p+k4pp;

double k5=kcaoff;

double k6=h6*cai*kcaon;

double k7=h5*h2*wna;

double k8=h8*h11*wna;

double x1=k2*k4*(k7+k6)+k5*k7*(k2+k3);

double x2=k1*k7*(k4+k5)+k4*k6*(k1+k8);

double x3=k1*k3*(k7+k6)+k8*k6*(k2+k3);

double x4=k2*k8*(k4+k5)+k3*k5*(k1+k8);

double E1=x1/(x1+x2+x3+x4);

double E2=x2/(x1+x2+x3+x4);

double E3=x3/(x1+x2+x3+x4);

double E4=x4/(x1+x2+x3+x4);

double KmCaAct=150.0e-6;

double allo=1.0/(1.0+pow(KmCaAct/cai,2.0));

double zna=1.0;

double JncxNa=3.0*(E4*k7-E1*k8)+E3*k4pp-E2*k3pp;

double JncxCa=E2*k2-E1*k1;

double Gncx=0.0008;

if (celltype==1)

{

Gncx*=1.1;

}

if (celltype==2)

{

Gncx*=1.4;

}

INaCa_i=0.8*Gncx*allo*(zna*JncxNa+zca*JncxCa);

h1=1+nass/kna3*(1+hna);

h2=(nass*hna)/(kna3*h1);

h3=1.0/h1;

h4=1.0+nass/kna1*(1+nass/kna2);

h5=nass*nass/(h4*kna1*kna2);

h6=1.0/h4;

h7=1.0+nao/kna3*(1.0+1.0/hna);

h8=nao/(kna3*hna*h7);

h9=1.0/h7;

h10=kasymm+1.0+nao/kna1*(1+nao/kna2);

h11=nao*nao/(h10*kna1*kna2);

h12=1.0/h10;

k1=h12*cao*kcaon;

k2=kcaoff;

k3p=h9*wca;

k3pp=h8*wnaca;

k3=k3p+k3pp;

k4p=h3*wca/hca;

k4pp=h2*wnaca;

k4=k4p+k4pp;

k5=kcaoff;

k6=h6*cass*kcaon;

k7=h5*h2*wna;

k8=h8*h11*wna;

x1=k2*k4*(k7+k6)+k5*k7*(k2+k3);

x2=k1*k7*(k4+k5)+k4*k6*(k1+k8);

x3=k1*k3*(k7+k6)+k8*k6*(k2+k3);

x4=k2*k8*(k4+k5)+k3*k5*(k1+k8);

E1=x1/(x1+x2+x3+x4);

E2=x2/(x1+x2+x3+x4);

E3=x3/(x1+x2+x3+x4);

E4=x4/(x1+x2+x3+x4);

KmCaAct=150.0e-6;

allo=1.0/(1.0+pow(KmCaAct/cass,2.0));

JncxNa=3.0*(E4*k7-E1*k8)+E3*k4pp-E2*k3pp;

JncxCa=E2*k2-E1*k1;

INaCa_ss=0.2*Gncx*allo*(zna*JncxNa+zca*JncxCa);

INaCa=INaCa_i+INaCa_ss;

double k1p=949.5;

double k1m=182.4;

double k2p=687.2;

double k2m=39.4;

k3p=1899.0;

double k3m=79300.0;

k4p=639.0;

double k4m=40.0;

double Knai0=9.073;

double Knao0=27.78;

double delta=-0.1550;

double Knai=Knai0*exp((delta*v*F)/(3.0*R*T));

double Knao=Knao0*exp(((1.0-delta)*v*F)/(3.0*R*T));

double Kki=0.5;

double Kko=0.3582;

double MgADP=0.05;

double MgATP=9.8;

double Kmgatp=1.698e-7;

double H=1.0e-7;

double eP=4.2;

double Khp=1.698e-7;

double Knap=224.0;

double Kxkur=292.0;

double P=eP/(1.0+H/Khp+nai/Knap+ki/Kxkur);

double a1=(k1p*pow(nai/Knai,3.0))/(pow(1.0+nai/Knai,3.0)+pow(1.0+ki/Kki,2.0)-1.0);

double b1=k1m*MgADP;

double a2=k2p;

double b2=(k2m*pow(nao/Knao,3.0))/(pow(1.0+nao/Knao,3.0)+pow(1.0+ko/Kko,2.0)-1.0);

double a3=(k3p*pow(ko/Kko,2.0))/(pow(1.0+nao/Knao,3.0)+pow(1.0+ko/Kko,2.0)-1.0);

double b3=(k3m*P*H)/(1.0+MgATP/Kmgatp);

double a4=(k4p*MgATP/Kmgatp)/(1.0+MgATP/Kmgatp);

double b4=(k4m*pow(ki/Kki,2.0))/(pow(1.0+nai/Knai,3.0)+pow(1.0+ki/Kki,2.0)-1.0);

x1=a4*a1*a2+b2*b4*b3+a2*b4*b3+b3*a1*a2;

x2=b2*b1*b4+a1*a2*a3+a3*b1*b4+a2*a3*b4;

x3=a2*a3*a4+b3*b2*b1+b2*b1*a4+a3*a4*b1;

x4=b4*b3*b2+a3*a4*a1+b2*a4*a1+b3*b2*a1;

E1=x1/(x1+x2+x3+x4);

E2=x2/(x1+x2+x3+x4);

E3=x3/(x1+x2+x3+x4);

E4=x4/(x1+x2+x3+x4);

double zk=1.0;

double JnakNa=3.0*(E1*a3-E2*b3);

double JnakK=2.0*(E4*b1-E3*a1);

double Pnak=30;

if (celltype==1)

{

Pnak*=0.9;

}

if (celltype==2)

{

Pnak*=0.7;

}

INaK=Pnak*(zna*JnakNa+zk*JnakK);

double xkb=1.0/(1.0+exp(-(v-14.48)/18.34));

double GKb=0.003;

IKb=GKb*xkb*(v-EK);

double PNab=3.75e-10;

INab=PNab*vffrt*(nai*exp(vfrt)-nao)/(exp(vfrt)-1.0);

double PCab=2.5e-8;

ICab=PCab*4.0*vffrt*(cai*exp(2.0*vfrt)-0.341*cao)/(exp(2.0*vfrt)-1.0);

double GpCa=0.0005;

IpCa=GpCa*cai/(0.0005+cai);

}

void FBC()

{

double CaMKb=CaMKo*(1.0-CaMKt)/(1.0+KmCaM/cass);

CaMKa=CaMKb+CaMKt;

CaMKt+=dt*(aCaMK*CaMKb*(CaMKb+CaMKt)-bCaMK*CaMKt);

JdiffNa=(nass-nai)/2.0;

JdiffK=(kss-ki)/2.0;

Jdiff=(cass-cai)/0.2;

double bt=4.75;

double a_rel=0.5*bt;

double Jrel_inf=a_rel*(-ICaL)/(1.0+pow(1.5/cajsr,8.0));

if (celltype==2)

{

Jrel_inf*=1.7;

}

double tau_rel=bt/(1.0+0.0123/cajsr);

if (tau_rel<0.005)

{

tau_rel=0.005;

}

Jrelnp=Jrel_inf-(Jrel_inf-Jrelnp)*exp(-dt/tau_rel);

double btp=1.25*bt;

double a_relp=0.5*btp;

double Jrel_infp=a_relp*(-ICaL)/(1.0+pow(1.5/cajsr,8.0));

if (celltype==2)

{

Jrel_infp*=1.7;

}

double tau_relp=btp/(1.0+0.0123/cajsr);

if (tau_relp<0.005)

{

tau_relp=0.005;

}

Jrelp=Jrel_infp-(Jrel_infp-Jrelp)*exp(-dt/tau_relp);

double fJrelp=(1.0/(1.0+KmCaMK/CaMKa));

Jrel=(1.0-fJrelp)*Jrelnp+fJrelp*Jrelp;

double Jupnp=0.004375*cai/(cai+0.00092);

double Jupp=2.75*0.004375*cai/(cai+0.00092-0.00017);

if (celltype==1)

{

Jupnp*=1.3;

Jupp*=1.3;

}

double fJupp=(1.0/(1.0+KmCaMK/CaMKa));

Jleak=0.0039375*cansr/15.0;

Jup=(1.0-fJupp)*Jupnp+fJupp*Jupp-Jleak;

Jtr=(cansr-cajsr)/100.0;

nai+=dt*(-(INa+INaL+3.0*INaCa_i+3.0*INaK+INab)*Acap/(F*vmyo)+JdiffNa*vss/vmyo);

nass+=dt*(-(ICaNa+3.0*INaCa_ss)*Acap/(F*vss)-JdiffNa);

ki+=dt*(-(Ito+IKr+IKs+IK1+IKb+Ist-2.0*INaK)*Acap/(F*vmyo)+JdiffK*vss/vmyo);

kss+=dt*(-(ICaK)*Acap/(F*vss)-JdiffK);

double Bcai;

if (celltype==1)

{

Bcai=1.0/(1.0+1.3*cmdnmax*kmcmdn/pow(kmcmdn+cai,2.0)+trpnmax*kmtrpn/pow(kmtrpn+cai,2.0));

}

else

{

Bcai=1.0/(1.0+cmdnmax*kmcmdn/pow(kmcmdn+cai,2.0)+trpnmax*kmtrpn/pow(kmtrpn+cai,2.0));

}

cai+=dt*(Bcai*(-(IpCa+ICab-2.0*INaCa_i)*Acap/(2.0*F*vmyo)-Jup*vnsr/vmyo+Jdiff*vss/vmyo));

double Bcass=1.0/(1.0+BSRmax*KmBSR/pow(KmBSR+cass,2.0)+BSLmax*KmBSL/pow(KmBSL+cass,2.0));

cass+=dt*(Bcass*(-(ICaL-2.0*INaCa_ss)*Acap/(2.0*F*vss)+Jrel*vjsr/vss-Jdiff));

cansr+=dt*(Jup-Jtr*vjsr/vnsr);

double Bcajsr=1.0/(1.0+csqnmax*kmcsqn/pow(kmcsqn+cajsr,2.0));

cajsr+=dt*(Bcajsr*(Jtr-Jrel));

}

void voltage()

{

v+=-dt*(INa+INaL+Ito+ICaL+ICaNa+ICaK+IKr+IKs+IK1+INaCa+INaK+INab+IKb+IpCa+ICab+Ist);

}

void stimulus()

{

if ((t>(start+n*CL) && t<(start+duration+n*CL-dt)))

{

if (Ist==0)

{

vrest=v;

}

Ist=amp;

}

else if (t>(start+duration+n*CL-dt))

{

Ist=0.0;

n=n+1;

}

}

void dVdt_APD()

{

vdot_old=vdot;

vdot=(v-vo)/dt;

if (APD_flag==0 && v>-40 && vdot<vdot_old)

{

vdot_max=vdot_old;

t_vdot_max=t-dt;

APD_flag=1;

}

if (APD_flag==1 && v<0.9*vrest)

{

APD=t-t_vdot_max;

APD_flag=0;

}

}

References

1. O'Hara T, Virag L, Varro A, Rudy Y (2011) Simulation of the undiseased human cardiac

ventricular action potential: model formulation and experimental validation. PLoS Comput Biol 7: e1002061.
